# Effects of Graft Bending Angle on the Tensile Behavior of Bone Tunnel-Graft Constructs for Anterior Cruciate Ligament Reconstruction Using Bone-Patellar Tendon–Bone Graft

**DOI:** 10.3390/bioengineering13070752

**Published:** 2026-06-26

**Authors:** Satoshi Yamakawa, Konsei Shino, Tomoki Ohori, Ken Nakata

**Affiliations:** 1 Department of Health and Sport Sciences, Medicine for Sports and Performing Arts, Graduate School of Medicine, The University of Osaka, Osaka 565-0871, Japan; 2 Sports Orthopaedic Center, Yukioka Hospital, 2-2-3 Ukita, Osaka 530-0021, Japan; 3 Department of Orthopaedic Surgery, Graduate School of Medicine, The University of Osaka, Osaka 565-0871, Japan

**Keywords:** bone–patellar tendon–bone graft, anterior cruciate ligament reconstruction, graft bending angle, mechanical properties, tendon insertion angle

## Abstract

Bone–patellar tendon–bone (BPTB) grafts used in anterior cruciate ligament reconstruction bend at the femoral tunnel aperture, forming a graft bending angle (GBA). However, the mechanical effects of GBA-related angled loading on bone–BPTB graft constructs remain unclear. This study investigated the effects of GBA and cyclic tensile loading on stiffness and elongation using porcine BPTB grafts. Fourteen specimens were assigned to tibial bone plug constructs (Type T, *n* = 7) or patellar bone plug constructs (Type P, *n* = 7). Each construct was cyclically loaded between 10 and 50 N for 50 cycles under GBA conditions of 0°, 30°, 60°, and 90°. Regardless of GBA condition, cyclic loading produced substantial increases in stiffness and elongation during the first 10 cycles, followed by smaller changes. Stiffness tended to decrease under greater GBA conditions, particularly in Type T constructs, where 60° and 90° reduced stiffness and increased elongation. Type P stiffness showed little dependence on GBA. At 0° and 30°, Type P showed lower stiffness and greater elongation than Type T. These findings indicate that bone–BPTB graft tensile behavior is governed by GBA-related loading geometry and bone plug–tendon junction morphology.

## 1. Introduction

**Figure 1:**
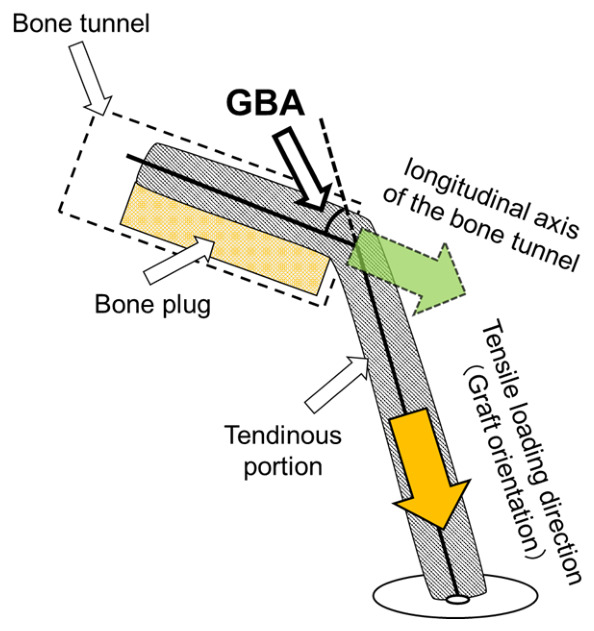
Schematic illustration and definition of the graft bending angle (GBA) in a bone–BPTB graft construct. The GBA was defined as the angle formed at the intra-articular aperture of the femoral tunnel between the intraosseous (bone plug) and intra-articular (tendinous portion) portions of the graft. The graft bends at the femoral tunnel aperture, where the bone plug–tendon junction is positioned. The yellow arrow indicates the tensile loading direction, and the green arrow indicates the long axis of the bone tunnel (plug). BPTB, bone–patellar tendon–bone; GBA, graft bending angle.

Anterior cruciate ligament (ACL) reconstruction is performed to restore knee stability by replacing the ruptured ligament with a graft routed through femoral and tibial bone tunnels [1,2,3]. The mechanical environment of the implanted graft is therefore influenced not only by the intrinsic properties of the graft tissue but also by the bone-graft construct. Bone–patellar tendon–bone (BPTB) grafts are commonly used because they allow bone-to-bone healing, provide secure initial fixation, and are supported by favorable clinical outcomes and graft failure data in recent systematic reviews [3,4,5,6,7,8,9,10]. Because the bone–tendon junction of the graft is generally positioned near the intra-articular aperture of the femoral tunnel, the graft changes direction at the bone plug–tendon junction with the intra-articular aperture acting as a fulcrum, forming the graft bending angle (GBA, Figure 1) [11,12,13,14,15]. This geometric configuration may increase local stress at the tunnel aperture and alter load transmission through the bone plug–tendon junction.

Previous studies have suggested that GBA may affect postoperative graft behavior through several mechanisms, including increased local stress at the tunnel aperture [16,17], altered graft–tunnel contact [18], impaired graft maturation [19,20], and delayed graft healing [21]. Most of these studies have evaluated GBA using clinical imaging, postoperative tunnel–graft morphology, or computational analyses of stress concentration. Although these approaches have provided important information regarding the clinical relevance of GBA, they do not directly characterize how GBA-related angled loading alters the mechanical response of the bone–BPTB graft construct during cyclic tensile loading.

The mechanical properties of BPTB grafts have generally been characterized using uniaxial tensile testing, in which the long axes of the bone plug and tendon segment are aligned collinearly and tensile load is applied along this common axis [22,23,24]. Although such tests provide essential information on the mechanical properties of the graft, they do not account for the angled loading condition imposed on the bone–BPTB graft construct by GBA. Thus, the mechanical behavior of the construct is influenced not only by the intrinsic properties of the BPTB graft but also by the GBA.

In ACL reconstruction, grafts are pre-tensioned or cyclically loaded before final fixation to reduce subsequent graft elongation and stabilize graft tension [25,26]. Mechanically, cyclic tensile loading may increase stiffness by removing initial slack and promoting structural settling within the bone–BPTB graft construct. At the same time, the cyclic loading may induce construct elongation relative to the initial state, with most lengthening occurring during the early loading cycles and then converging thereafter. However, it remains unclear how GBA-related angled loading affects the stiffening, elongation, and stabilization responses of bone–BPTB graft constructs during cyclic tensile loading. To address this gap, an experimental model that can systematically control GBA is needed to evaluate how graft bending geometry influences construct stiffness and elongation during cyclic tensile loading.

This study aimed to determine how GBA-related angled loading and cyclic tensile loading affect the stiffness and elongation of the bone–BPTB graft constructs. We hypothesized that stiffness would increase during the early loading cycles and subsequently stabilize, whereas elongation would increase mainly during the early loading cycles before reaching a plateau. We further hypothesized that greater GBA conditions would reduce stiffness and increase elongation because of less favorable load transmission at the bone plug–tendon junction of the BPTB graft.

## 2. Materials and Methods

### 2.1. Study Design

This study was designed to evaluate the effects of GBA-related angled loading and cyclic tensile loading on the mechanical behavior of the bone–BPTB graft constructs. Because a BPTB graft includes two types of bone–tendon junction, corresponding to the tibial and patellar plugs [27,28], two construct types were prepared. Type T constructs were defined as constructs in which the tibial bone plug was mounted in the angle-adjustable jig, whereas Type P constructs were defined as constructs in which the patellar bone plug was mounted in the jig. In both construct types, the mounted bone plug was subjected to GBA-related angled loading.

The primary purpose of the experiment was to determine how GBA conditions affect construct stiffness and elongation during cyclic tensile loading. As a secondary purpose, Type T and Type P constructs were compared because the gross morphology appeared to differ between the two bone–tendon junctions. This comparison was intended to examine which bone plug–tendon junction should be positioned at the femoral tunnel aperture from the perspective of the mechanical response to GBA-related angled loading.

**Figure 2:**
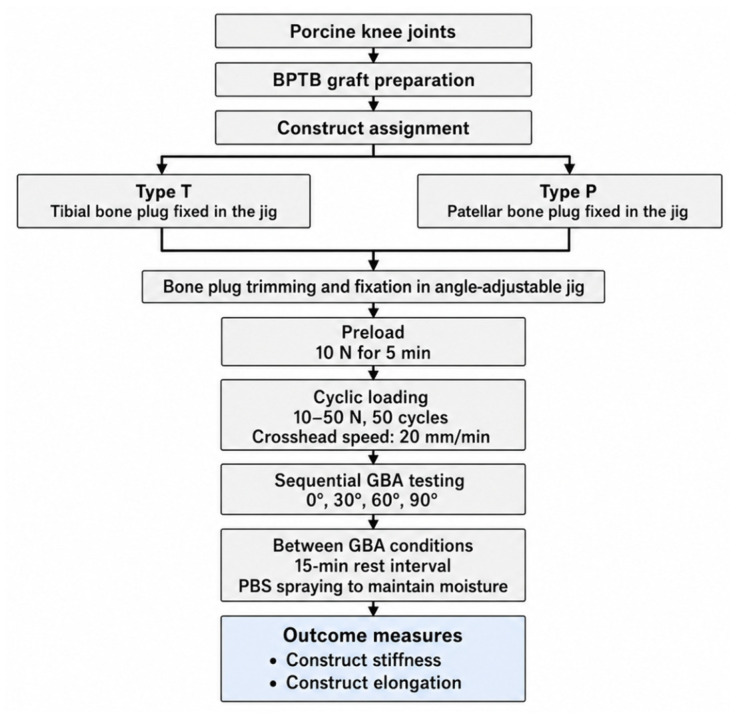
Schematic overview of the experimental design. Porcine bone–patellar tendon–bone (BPTB) grafts were prepared and assigned to Type T or Type P constructs, which indicate constructs tested with the tibial and patellar bone plugs, respectively. Each construct was tested sequentially under graft bending angle (GBA) conditions of 0°, 30°, 60°, and 90°. At each GBA condition, specimens underwent a 10 N preload for 5 min followed by cyclic loading between 10 and 50 N for 50 cycles at 20 mm/min. A 15 min rest interval with phosphate-buffered saline (PBS) spraying was applied between GBA conditions. Construct stiffness and elongation were calculated as outcome measures. BPTB, bone–patellar tendon–bone; GBA, graft bending angle; PBS, phosphate-buffered saline.

The experimental workflow, including specimen preparation, construct assignment, GBA conditions, cyclic loading protocol, rest intervals, and outcome measurements, is provided in Figure 2.

### 2.2. Specimen Preparation

Porcine BPTB grafts were prepared from immature knee joints of 6-month-old pigs (*n* = 14). The specimens were assigned to one of two construct groups according to which bone plug was mounted in the angle-adjustable jig: Type T constructs (*n* = 7) and Type P constructs (*n* = 7). The sample size was selected based on previous ex vivo biomechanical studies using similar graft constructs and cyclic loading protocols, as well as the practical availability of porcine specimens. In addition, because each specimen was tested sequentially under all GBA conditions, the repeated-measures design was used to reduce the influence of inter-specimen variability when evaluating GBA-dependent changes in stiffness and elongation.

**Figure 3:**
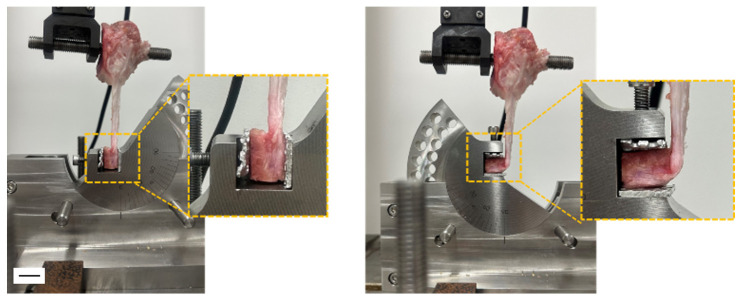
Custom-made angle-adjustable jig used to reproduce graft bending angle (GBA) conditions in bone–BPTB graft constructs. Representative photographs show the bone–BPTB graft construct mounted in the jig at GBA 0° (**left**) and GBA 90° (**right**). The bone plug was secured in the angle-adjustable jig, and the opposite bony end was connected to the upper grip of the testing machine. The enlarged views show the bone plug–tendon junction positioned at the jig side, where GBA-related angled loading was applied. BPTB, bone–patellar tendon–bone; GBA, graft bending angle. Scale bars indicate 10 mm.

For each specimen, the bone plug mounted in the jig was trimmed to a rectangular shape measuring 15 mm in length, 10 mm in width, and 5 mm in thickness. The bone plug was secured to a custom-made angle-adjustable jig that allowed the GBA to be adjusted relative to the tensile loading direction (Figure 3). The opposite bony end was drilled with an 8 mm-diameter hole and connected to the upper grip of the testing machine using a stainless-steel bolt.

### 2.3. Mechanical Testing Protocol

Mechanical testing was performed using a material testing machine (AutoGraph, Shimadzu, Kyoto, Japan). Before cyclic testing, each specimen was mounted at a GBA of 0° and held under a preload of 10 N for 5 min. The 10 N preload was selected to remove initial slack in the construct and to establish a reproducible baseline displacement under a low initial tensile load. Cyclic tensile loading was then applied between 10 and 50 N at a crosshead speed of 20 mm/min for 50 cycles. The cyclic loading range of 10–50 N was used to evaluate the initial cyclic settling behavior of the bone–BPTB graft construct while avoiding excessive loading or gross damage during repeated testing under multiple GBA conditions. This loading protocol was intended to characterize the early mechanical response of the construct under controlled low-load cyclic tension, rather than to reproduce physiological ACL graft forces in vivo.

Each specimen was tested sequentially at GBA values of 0°, 30°, 60°, and 90°. After completion of testing at each angle, except for the final 90° condition, the specimen was kept moist and allowed to rest for 15 min before the jig was adjusted to the next GBA condition. Mechanical testing was performed at room temperature. Because each cyclic loading test lasted approximately 5 min, the specimens were sprayed with phosphate-buffered saline (PBS) before and after each loading test and during the 15 min rest intervals to maintain a moist condition and prevent visible surface drying. Ambient humidity was not actively controlled, and specimen water content was not quantitatively measured before or after testing. The same loading protocol was applied at each angle in both the Type T and P constructs.

Tensile force and crosshead displacement were recorded continuously throughout testing. For stiffness analysis, the loading portion of each selected cycle was used. Construct stiffness was defined as the slope of the load–displacement curve between 30 and 40 N and was expressed in N/mm. Stiffness was calculated for the 1st, 10th, 20th, 30th, 40th, and 50th loading cycles.

Construct elongation during cyclic loading was evaluated at 10-cycle intervals. For each GBA condition, the displacement at 10 N before cyclic loading was defined as the baseline displacement. Elongation at the 10th, 20th, 30th, 40th, and 50th loading cycles was calculated as the increase in displacement at 10 N relative to this baseline value.

### 2.4. Statistical Analysis

Data are presented as mean ± standard deviation. The effects of construct type, GBA, and loading cycle on construct stiffness and elongation were analyzed using mixed-design ANOVA. Construct type was treated as a between-specimen factor, whereas GBA and loading cycle were treated as within-specimen factors because measurements at different GBA conditions and loading cycles were obtained from the same specimen. When significant main effects or interactions were detected, post hoc pairwise comparisons were performed with Bonferroni correction. For comparisons among GBA conditions within the same construct type, paired comparisons were used. For comparisons between Type T and P constructs at the same GBA condition, independent comparisons were used. Statistical significance was set at *p* < 0.05.

## 3. Results

**Figure 4:**
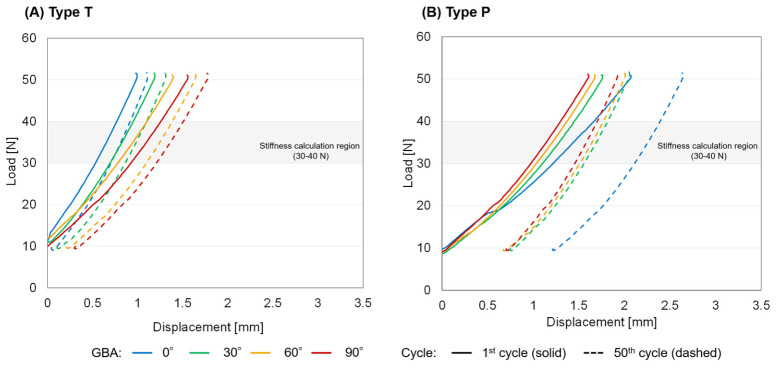
Representative loading portions of the load–displacement curves during cyclic tensile loading under different graft bending angle (GBA) conditions. (**A**) Type T constructs. (**B**) Type P constructs. Solid and dashed lines indicate the first and 50th loading cycles, respectively. Line colors indicate GBA conditions of 0°, 30°, 60°, and 90°. For each GBA condition, displacement was normalized to zero at the point closest to 10 N in the first loading cycle, and only the loading portion from that point onward is shown. The shaded region indicates the 30–40 N range used for stiffness calculation. GBA, graft bending angle.

**Figure 5:**
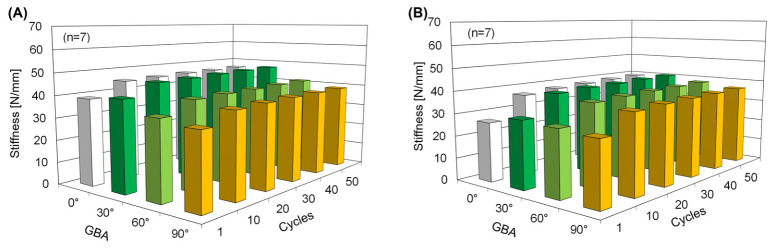
Changes in construct stiffness during cyclic tensile loading under different graft bending angle (GBA) conditions. Three-dimensional bar graphs show stiffness values of the bone–BPTB graft constructs at the 1st, 10th, 20th, 30th, 40th, and 50th loading cycles under each GBA condition. (**A**) Type T constructs. (**B**) Type P constructs. Bar colors correspond to the GBA conditions shown on the GBA axis (0°, 30°, 60°, and 90°). Stiffness was calculated from the linear region of the load–displacement curve between 30 and 40 N. GBA, graft bending angle; BPTB, bone–patellar tendon–bone.

**Figure 6:**
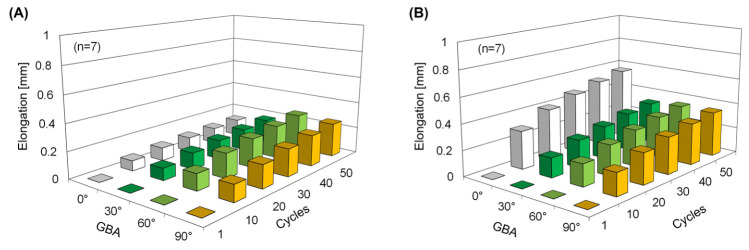
Changes in construct elongation during cyclic tensile loading under different graft bending angle (GBA) conditions. Three-dimensional bar graphs show elongation values of the bone–BPTB graft constructs at the 10th, 20th, 30th, 40th, and 50th loading cycles under each GBA condition. (**A**) Type T constructs; (**B**) Type P constructs. Bar colors correspond to the GBA conditions shown on the GBA axis (0°, 30°, 60°, and 90°). Elongation was calculated as the increase in the distance between fixation points at 10 N relative to the baseline value before cyclic loading. GBA, graft bending angle; BPTB, bone–patellar tendon–bone.

Across all GBA conditions, both Type T and Type P bone–BPTB graft constructs showed a similar cycle-dependent response during cyclic tensile loading (Figure 4, Figure 5 and Figure 6; Table 1 and Table 2). Representative loading portions of the load–displacement curves are shown in Figure 4. In both construct types, stiffness increased mainly during the first 10 loading cycles and then showed only small additional increases up to the 50th cycle. Construct elongation also increased relative to baseline across all GBA conditions, with the largest increase occurring by the 10th loading cycle followed by smaller additional increases thereafter. Thus, early increases and subsequent stabilization of stiffness and elongation were common responses regardless of GBA condition.

Overall, Type T constructs tended to show greater stiffness and smaller elongation than Type P constructs, although the magnitude of these construct-dependent differences varied according to GBA condition. With respect to GBA-related differences, greater GBA conditions, particularly 60° and 90°, were associated with lower stiffness and greater elongation. This angle-dependent response was more clearly observed in Type T constructs than in Type P constructs. In Type T constructs, stiffness was significantly lower under greater GBA conditions at several loading cycles, and elongation was significantly greater at 60° and 90° than at 0° throughout cyclic loading. These findings indicate that greater GBA conditions reduced stiffness and increased cumulative elongation in Type T constructs.

In Type P constructs, the GBA-dependent response was less pronounced. No significant differences in stiffness were observed among GBA conditions at any loading cycle, although mean stiffness tended to be lower under greater GBA conditions than at 30°. In addition, construct-dependent differences in stiffness and elongation were not significant at greater GBA conditions, except for lower stiffness in Type P constructs at 60° during the 1st loading cycle. These findings indicate that the construct-dependent mechanical advantage observed in Type T constructs became less apparent under greater GBA conditions.

Under smaller GBA conditions, particularly 0° and 30°, construct-dependent differences were more evident. Type T constructs showed a more favorable stiffness–elongation profile than Type P constructs, characterized by greater stiffness and smaller elongation. At 0° and 30°, elongation of Type P constructs was significantly greater than that of Type T constructs throughout cyclic loading, whereas stiffness differences between construct types were observed only at selected loading cycles. Within Type P constructs, elongation at 0° was significantly greater than that at 30° during the later loading cycles. Detailed values and statistical comparisons are provided in Table 1 and Table 2.

## 4. Discussion

The primary finding of this study was that cyclic tensile loading produced a common stabilization response in bone–BPTB graft constructs regardless of GBA condition. Under both the 0° GBA condition, which represents uniaxial loading, and GBA-related angled loading conditions, stiffness increased mainly during the first 10 loading cycles, while elongation also increased relative to baseline during the same early phase. Thereafter, both stiffness and elongation showed only small additional changes up to the 50th cycle in both Type T and Type P constructs. These findings indicate that cyclic tensile loading induced early construct settling as a common mechanical response, independent of loading angle. This early stabilization behavior is consistent with previous biomechanical studies showing that graft pre-tensioning or cyclic loading can reduce subsequent graft elongation and improve initial construct stability [25,26]. The present findings extend these observations by showing that a similar early stabilization response occurs not only under uniaxial loading but also under GBA-related angled loading, although the magnitudes of stiffness and elongation depend on GBA and construct type. From a mechanical standpoint, graft pre-tensioning or cyclic preconditioning with at least 10 loading cycles may therefore be useful before final fixation to promote stabilization of the bone–BPTB graft construct.

The present findings extend previous studies on GBA by experimentally demonstrating that GBA-related angled loading can alter the cyclic tensile behavior of bone–BPTB graft constructs. Previous clinical and imaging studies have emphasized the association of GBA with graft bending at the tunnel aperture [11,12,13,14,15], graft–tunnel interaction and tunnel widening [18], graft maturation [19,20], or graft healing [21], whereas computational and theoretical studies have suggested that greater graft bending may increase local stress concentration [16,17]. In contrast, the present study focused on the construct-level mechanical response under controlled GBA conditions and showed that greater GBA conditions tended to reduce stiffness and increase elongation during cyclic loading, particularly in Type T constructs. Thus, this study provides experimental evidence linking GBA-related loading geometry to measurable changes in stiffness and elongation of bone–BPTB graft constructs.

**Figure 7:**
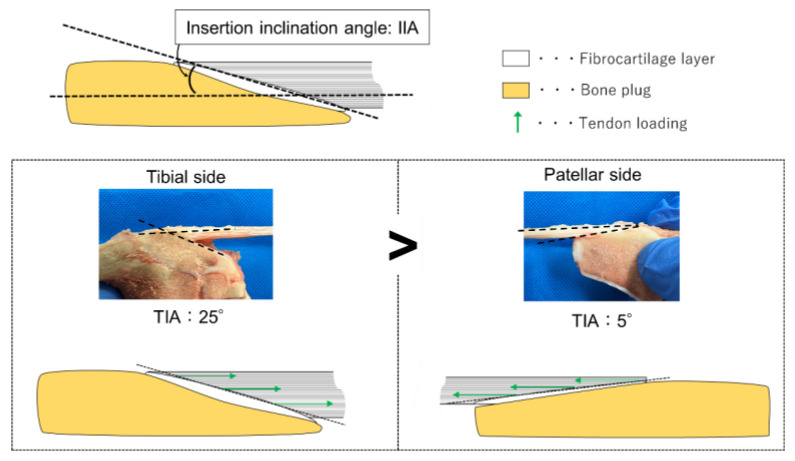
Representative measurements showed a larger TIA at the tibial-side junction, corresponding to the Type T construct, and a smaller TIA at the patellar-side junction, corresponding to the Type P construct. The schematic illustrates that differences in TIA may alter the local relationship between the applied tensile load direction and the tendon insertion surface, thereby potentially influencing load transfer through the bone plug–tendon junction.

Another important finding was that, although the early stabilization response was observed regardless of GBA condition, the magnitudes of stiffness and elongation varied depending on GBA and construct type. Across the tested conditions, Type T constructs generally tended to show greater stiffness and smaller elongation than Type P constructs, although statistically significant construct-type-dependent differences were mainly observed under smaller GBA conditions. These construct-dependent responses suggest that the effect of GBA was not determined by the bending angle alone. One possible explanation is the difference in insertion-site geometry, including the tendon insertion angle (TIA; Figure 7). In this study, TIA was defined as the sagittal-plane angle between the tendon insertion surface and the long axis of the bone plug. Representative measurements from specimens selected from the sample showed that TIA was larger in the tibial-side insertion than in the patellar-side insertion, with approximate values of 25° and 5°, respectively. These observations are consistent with previous anatomical findings [27,28] and support the concept that the tendon insertion surface may be oriented differently relative to the applied tensile load in Type T and Type P constructs. Therefore, even under the same GBA condition, the local load-transfer condition from the tendon to the bone plug may differ between the two constructs. However, TIA was not quantitatively measured in all specimens or incorporated as a biomechanical variable in the statistical analysis. Further studies with systematic TIA measurements and local deformation analyses are needed to clarify how insertion-site geometry contributes to load transfer and the construct-dependent mechanical responses observed in this study. When extrapolating these findings to human BPTB grafts, the observed Type T–Type P differences should also be interpreted in light of possible species- and maturity-related differences in tendon properties, bone quality, and insertion-site morphology.

As GBA increased, stiffness tended to decrease, and this angle-dependent response was most clearly observed in Type T constructs. Under greater GBA conditions (60° and 90°), Type T constructs showed reduced stiffness and greater elongation. These results suggest that greater GBA-related angled loading reduced the favorable load-transfer condition of the Type T construct. In contrast, Type P constructs showed no significant differences in stiffness among GBA conditions, although mean stiffness tended to be lower under greater GBA conditions than at 30°. Construct-dependent differences in stiffness and elongation were not observed at 60° or 90°, except for lower Type P stiffness at the 1st cycle at 60°. These findings suggest that the mechanical advantage associated with the greater TIA at the tibial insertion may be reduced when the GBA-imposed loading direction deviates substantially from the orientation of the tendon insertion surface.

Under smaller GBA conditions (0° and 30°), Type T constructs showed a more favorable stiffness–elongation profile than Type P constructs. At 0°, stiffness of Type P constructs was significantly lower than that of Type T constructs from the 1st to the 40th loading cycle, and at 30° the difference was significant at the 1st cycle. In addition, elongation of Type P constructs was significantly greater than that of Type T constructs at both 0° and 30° throughout the cyclic loading protocol. One possible mechanism is that the smaller TIA at the patellar-side insertion produces a less favorable orientation of the tendon insertion surface relative to the applied tensile load. This may increase local settling or shear-related deformation within the fibrocartilaginous insertion region, resulting in lower stiffness and greater elongation in Type P constructs. However, because insertion-site deformation was not directly quantified, this mechanism should be interpreted as a proposed explanation rather than direct evidence.

Taken together, these findings suggest that the tensile behavior of bone–BPTB graft constructs is governed by the relationship between GBA-related loading geometry and bone plug–tendon junction morphology. GBA determines the macroscopic loading direction, whereas the junctional morphology, particularly the orientation of the tendon insertion surface represented by TIA, influences local load transfer from the tendon to the bone plug. Thus, BPTB grafts should be considered not only as bone-to-bone fixation grafts but also as junctional load-transfer constructs. Clinically, positioning the tibial-side bone plug–tendon junction at the femoral tunnel side may be mechanically advantageous when excessive GBA is avoided, whereas greater GBA conditions may reduce this potential advantage. Therefore, surgical strategies that preserve favorable load transfer at the bone plug–tendon junction while minimizing excessive GBA may help maximize the mechanical benefit of BPTB grafts, particularly in procedures with relatively small expected GBA, such as over-the-top or inlay techniques [29,30].

From a practical biomechanical perspective, these findings also suggest that tunnel aperture geometry, graft routing, and bone plug orientation should be evaluated together when considering graft design and fixation strategies, because the mechanical response of the graft may depend on both the direction of bending and the side of the bone plug–tendon junction subjected to angled loading. In addition, the angle-adjustable testing approach used in this study provides a simple experimental model for evaluating early cyclic settling, stiffness, and elongation under controlled GBA conditions, and may serve as a basis for future robotic or simulator-based testing systems in which graft bending angle, tensile load, and cyclic loading history can be controlled independently. Although the present results do not define specific postoperative rehabilitation limits, they suggest that early cyclic loading and construct settling should be considered when evaluating the mechanical safety of BPTB graft constructs, particularly under conditions involving greater graft bending. Because this study was performed under simplified ex vivo low-load conditions using a 10–50 N cyclic loading range, these clinical and biomechanical implications should be interpreted cautiously and validated under higher and more physiological loading and fixation conditions before being translated into specific surgical or rehabilitation recommendations.

This study has several limitations. First, the sample size was relatively small, with seven specimens in each construct group. Although the repeated-measures design reduced inter-specimen variability, the study may have been underpowered to detect smaller differences or interaction effects between construct type, GBA condition, and loading cycle. Second, immature porcine BPTB grafts were used; therefore, the findings may not directly represent the mechanical behavior of human BPTB grafts. Species- and maturity-related differences in tendon structure, bone quality, and tendon insertion morphology should be considered. Third, cyclic tensile loading was limited to 10–50 N, and subsequent failure testing was not performed. This low-load protocol was selected to evaluate initial cyclic settling while avoiding gross damage during repeated testing at multiple GBA conditions. However, the present study does not provide information regarding ultimate failure load, failure mode, or construct durability under higher or more physiological loading conditions. Therefore, the effects of GBA-related angled loading may differ under higher loads, and future studies should combine cyclic loading with subsequent failure testing to better evaluate construct safety and durability. In addition, because GBA conditions were tested sequentially in the same specimens, the possible effects of prior cyclic loading and hydration-related changes cannot be completely excluded, although rest intervals and PBS spraying were applied between conditions. Fourth, the experimental setup simplified the mechanical environment of ACL reconstruction. The constructs were tested using a custom angle-adjustable jig rather than an actual femoral tunnel, and tunnel wall contact, fixation devices, bone tunnel geometry, and surrounding joint structures were not reproduced. Future studies using histological assessment and more anatomically representative fixation conditions are needed. Fifth, local strain, stress distribution, and insertion-site deformation at the bone plug–tendon junction were not directly measured. Because the bone plug was compressed within the jig and the visible tendon-fiber region near the insertion site was limited, particularly in Type P constructs, direct application of digital image correlation to the present setup would not have allowed reliable comparison of local deformation across specimens and construct types. Therefore, the proposed load-transfer mechanism should be interpreted as a hypothesis rather than direct evidence. Future studies using larger sample sizes, more physiological fixation and loading conditions, histological assessment, and local deformation analyses such as digital image correlation, strain measurement, or finite element modeling are needed to confirm the present findings and clarify the tissue-level mechanisms underlying GBA-related angled loading.

## 5. Conclusions

(1) Cyclic tensile loading on bone–BPTB graft constructs produced substantial increases in both stiffness and elongation during the first 10 loading cycles regardless of GBA condition. Thereafter, only small additional changes were observed up to the 50th cycle.
(2) As GBA increased, stiffness tended to decrease. Under greater GBA conditions (60° and 90°), Type T constructs showed reduction in stiffness and increase in elongation, whereas stiffness in Type P constructs was less dependent on GBA. Under smaller GBA conditions (0° and 30°), Type P constructs showed lower stiffness and greater elongation than Type T constructs, indicating that Type T constructs had a more favorable stiffness–elongation profile when GBA was smaller.

## Figures and Tables

**Table 1:** Construct stiffness during cyclic tensile loading under each graft bending angle (GBA) condition. Values are presented as mean ± SD. Stiffness was calculated from the linear region of the load–displacement curve between 30 and 40 N for each loading cycle. Type T, tibial bone plug construct; Type P, patellar bone plug construct. GBA, graft bending angle; BPTB, bone–patellar tendon–bone.

Construct Type	GBA	1st Cycle (N/mm)	10th Cycle (N/mm)	20th Cycle (N/mm)	30th Cycle (N/mm)	40th Cycle (N/mm)	50th Cycle (N/mm)
Type T	0°	38.9 ± 3.4	45.0 ± 2.2	45.4 ± 2.2	45.9 ± 2.1	46.0 ± 2.2	46.3 ± 2.3
	30°	40.6 ± 3.8	45.8 ± 4.4	46.1 ± 4.5	46.6 ± 4.6	47.0 ± 4.5	47.2 ± 4.7
	60°	35.0 ± 4.4	40.1 ± 5.2 ^†^	40.6 ± 5.4	40.7 ± 5.3 ^†^	41.1 ± 5.5 ^†^	41.3 ± 5.6 ^†^
	90°	33.0 ± 4.8	37.9 ± 5.0 ^†^	38.4 ± 5.3	38.7 ± 5.5	38.7 ± 5.5 ^†^	38.9 ± 5.5 ^†^
Type P	0°	26.6 ± 2.9 ^‡^	36.6 ± 1.9 ^‡^	37.7 ± 1.7 ^‡^	38.3 ± 1.8 ^†^	38.8 ± 1.8 ^‡^	39.2 ± 1.9
	30°	30.2 ± 2.1 ^‡^	39.1 ± 2.1	39.7 ± 2.5	39.9 ± 2.3	40.3 ± 2.5	40.4 ± 2.5
	60°	29.2 ± 2.2 ^‡^	37.0 ± 3.0	37.6 ± 3.3	37.9 ± 3.2	38.1 ± 3.2	38.3 ± 3.3
	90°	28.0 ± 3.2	35.5 ± 3.9	36.1 ± 4.0	36.4 ± 4.0	36.5 ± 4.0	36.6 ± 4.1

Values are presented as mean ± SD. ^†^ Significantly different from 30° within the same construct at the same loading cycle (adjusted *p* < 0.05). ^‡^ Significantly different from the Type T construct at the same GBA and loading cycle (adjusted *p* < 0.05). Type T, tibial-side bone plug construct; Type P, patellar-side bone plug construct. GBA, graft bending angle; BPTB, bone–patellar tendon–bone.

**Table 2:** Construct elongation during cyclic tensile loading under each graft bending angle (GBA) condition. Values are presented as mean ± SD. Elongation was calculated as the increase in the distance between fixation points at 10 N relative to the baseline value before cyclic loading for each GBA condition. Values at the 10th, 20th, 30th, 40th, and 50th loading cycles represent cumulative elongation relative to baseline. Type T, tibial-side bone plug construct; Type P, patellar-side bone plug construct. GBA, graft bending angle; BPTB, bone–patellar tendon–bone.

Construct Type	GBA	10th Cycle (mm)	20th Cycle (mm)	30th Cycle (mm)	40th Cycle (mm)	50th Cycle (mm)
Type T	0°	0.076 ± 0.021	0.099 ± 0.019	0.110 ± 0.025	0.120 ± 0.023	0.127 ± 0.026
	30°	0.090 ± 0.029	0.123 ± 0.041	0.146 ± 0.041	0.163 ± 0.050	0.176 ± 0.054
	60°	0.123 ± 0.022 ^†^	0.186 ± 0.054 ^†^	0.219 ± 0.059 ^†‡^	0.247 ± 0.067 ^†‡^	0.270 ± 0.073 ^†‡^
	90°	0.123 ± 0.026 ^†^	0.172 ± 0.033 ^†^	0.205 ± 0.034 ^†^	0.232 ± 0.035 ^†^	0.254 ± 0.037 ^†^
Type P	0°	0.297 ± 0.043 ^§^	0.403 ± 0.052 ^§^	0.468 ± 0.052 ^‡§^	0.520 ± 0.054 ^‡§^	0.560 ± 0.052 ^‡§^
	30°	0.153 ± 0.017 ^§^	0.218 ± 0.026 ^§^	0.257 ± 0.034 ^§^	0.298 ± 0.042 ^§^	0.323 ± 0.049 ^§^
	60°	0.174 ± 0.039	0.240 ± 0.046	0.285 ± 0.054	0.323 ± 0.060	0.349 ± 0.069
	90°	0.177 ± 0.034	0.244 ± 0.046	0.289 ± 0.056	0.323 ± 0.061	0.349 ± 0.072

Values are presented as mean ± SD. ^†^ Significantly different from 0° within the same construct at the same loading cycle (adjusted *p* < 0.05). ^‡^ Significantly different from 30° within the same construct at the same loading cycle (adjusted *p* < 0.05). ^§^ Significantly different from 0° within the Type T construct at the same GBA and loading cycle (adjusted *p* < 0.05). Elongation was calculated as the increase in the distance between fixation points at 10 N relative to the baseline value before cyclic loading. Type T, tibial-side bone plug construct; Type P, patellar-side bone plug construct. GBA, graft bending angle; BPTB, bone-patellar tendon-bone.

## Data Availability

The original contributions presented in this study are included in the article. Further inquiries can be directed to the corresponding author.
